# Quantification of colorimetric isothermal amplification on the smartphone and its open-source app for point-of-care pathogen detection

**DOI:** 10.1038/s41598-020-72095-3

**Published:** 2020-09-15

**Authors:** Huynh Quoc Nguyen, Van Dan Nguyen, Hau Van Nguyen, Tae Seok Seo

**Affiliations:** Department of Chemical Engineering, College of Engineering, Kyung Hee University, 1 Seochon-dong, Giheung-gu, Yongin-si, Gyeonggi-do 17140 Republic of Korea

**Keywords:** DNA, Computational biology and bioinformatics

## Abstract

The increasing risk of infectious pathogens, especially in the under-developed countries, is demanding the development of point-of-care (POC) nucleic acid testing in the low-resource setting conditions. Here, we describe a methodology for colorimetric quantitative analysis of nucleic acid using an easy-to-build smartphone-based platform, offering low-cost, portability, simplicity in operation, and user-friendliness. The whole system consists of a hand-held box equipped with a smartphone, a film heater, a white LED, a loop-mediated isothermal amplification (LAMP) chip, and a DC converter, and all the processes were powered by a portable battery of 5 V. Upon the amplification of the target gene by an Eriochrome Black T-mediated LAMP reaction, the color of the LAMP reaction was changed from violet to blue that was real-time recorded by a smartphone camera. To keep track of the progress of the color change, we developed a novel mobile app in which a hue value was accepted as an indicator for color transition and for determining the threshold time of the amplification reaction. A calibration curve could be generated by plotting the logarithm of the known concentration of the DNA templates versus the threshold time, and it can be used to predict the copy number of nucleic acids in the test samples. Thus, the proposed mobile platform can inform us of not only qualitative but also quantitative results of the pathogens. We believe that this advanced colorimetric approach and the mobile app can expand the potentials of the smartphone for the future POCT system in the bio-diagnostic fields.

## Introduction

Owing to the tremendous progress of Information & Communication Technology (ICT), the paradigm for medical diagnostics is shifting from the centralized medical centers to the individual home or ubiquitous places. Mobile communication via a smartphone or an internet of thing (IOT) device enables us to connect human network anytime anywhere, so the patient can have a person-to-person talk with doctors in case of need. Thus, the adoption of the ICT to the medical diagnostic fields is a future trend, and it will be the next-generation diagnostic technology. On the other hand, microfluidic technology has attracted huge attention due to its miniaturization, fast analysis time, low consumption of reagents, high integration, and full automation capability. Microfluidic based POCT systems have been proposed for genomic and cellular analysis and have reached a mature stage for commercialization. Such a POCT platform is important for the global healthcare monitoring, especially for those who are in need of prompt medical decisions^1^.

Recent progress of the POCT diagnostics moves towards the combination of ICT with the microfluidic device. The utilization of the smartphone for bio-diagnostics is rapidly growing due to the increasing popularity of the smartphone as well as the superb specification of the smartphone such as excellent CMOS sensors, computing capabilities, customized mobile apps, data transfer and storage^2^. Kanakasabapathy et al*.* uses the camera of smartphone and a series of optical accessories for CD4 detection under a bright field. Ganguli et al. employed a camera of a smartphone for identifying Zika virus in a whole blood sample by using a reverse-transcription loop-mediated isothermal amplification (RT-LAMP) and a fluorescence detection^3^. Paterson et al*.* uses the smartphone flash as an excitation source and a luminescent phosphor as a reporter for quantitative and rapid detection of human chorionic gonadotropin in urine^4^.

However, the previous studies used a limited function of the smartphone and its accessories, and few reports have been published for the genetic analysis, which is indispensable for the definite diagnosis of pathogens. Although polymerase chain reaction (PCR) is accepted as a gold standard method in clinical laboratories due to its high sensitivity and reliability, the precise control of temperature for thermal cycling and fluorescence-based detection system render the whole system bulky and expensive, making the PCR assay unsuitable for POC DNA testing. To address this issue, isothermal amplification techniques have been developed such as LAMP, rolling circle amplification, recombinase polymerase amplification, and helicase dependent amplification. The sophisticated design of primers and the use of enzymes are capable of gene amplification at one constant temperature, eliminating the effort for complicated temperature controller. Regarding the detection methods for the amplicons, electrochemical and colorimetric techniques were developed that are simpler and smaller than the fluorescence measurement equipment. In particular, the colorimetric detection is to use a metal indicator dye, for example, Eriochrome Black T (EBT) and hydroxy naphthol blue (HNB), or a pH-sensitive dye such as phenol red and NeuRed in the reaction cocktail, and the target gene amplification can be monitored simply by observing the color of the reaction mixture without need of laboratory-based instruments or expensive detectors/sensors. Considering all these factors, we believe that the best combination for the POCT is the isothermal amplification with the colorimetric detection. Recently, Park et al*.* report the colorimetric detection of LAMP by a lateral flow strip^5^, and Oh et al., Seo et al. and Nguyen et al., reported the colorimetric detection of LAMP by using EBT dyes^6–8^. A colorimetric LAMP assay is also commercialized by New England Biolabs Inc. using a pH-sensitive dye^9,10^. Although these combinations are convenient and rapid to inform us of the results at the endpoint, the quantification of the starting DNA copy number was not reported yet. The lack of the real-time digital interpretation of the color change and the subjective judgment of the color change may make it difficult to perform the quantitative analysis in the colorimetric assay. Since a few cells of pathogens can cause lethal effect on human health, the quantification of the pathogens is crucial. To achieve the quantification of the colorimetric loop-mediated isothermal amplification (qLAMP), several requirements should be met. First, unlike the conventional qPCR which plots the fluorescence intensity versus the number of thermal cycling, the colorimetric qLAMP should display the real-time amplification profile with the color value versus the reaction time. Second, the suitable colorimetric model should be established to express the degree of color change with high accuracy and robustness without interference of the external light conditions. Third, the miniaturized microsystem for qLAMP should be constructed for the POC DNA testing, which can be operated and monitored by a smartphone and its accessory. Lastly, the mobile app of the smartphone should be developed to plot the qLAMP profile and the calibration curve and produce the quantification data. In this study, we addressed the above requirements to propose a smartphone integrated POCT platform for nucleic acid quantification with a new colorimetric approach, simplicity of production and operation and a mobile app.

## Results

### Design of the integrated smartphone-based genetic analysis platform

**Figure 1:**
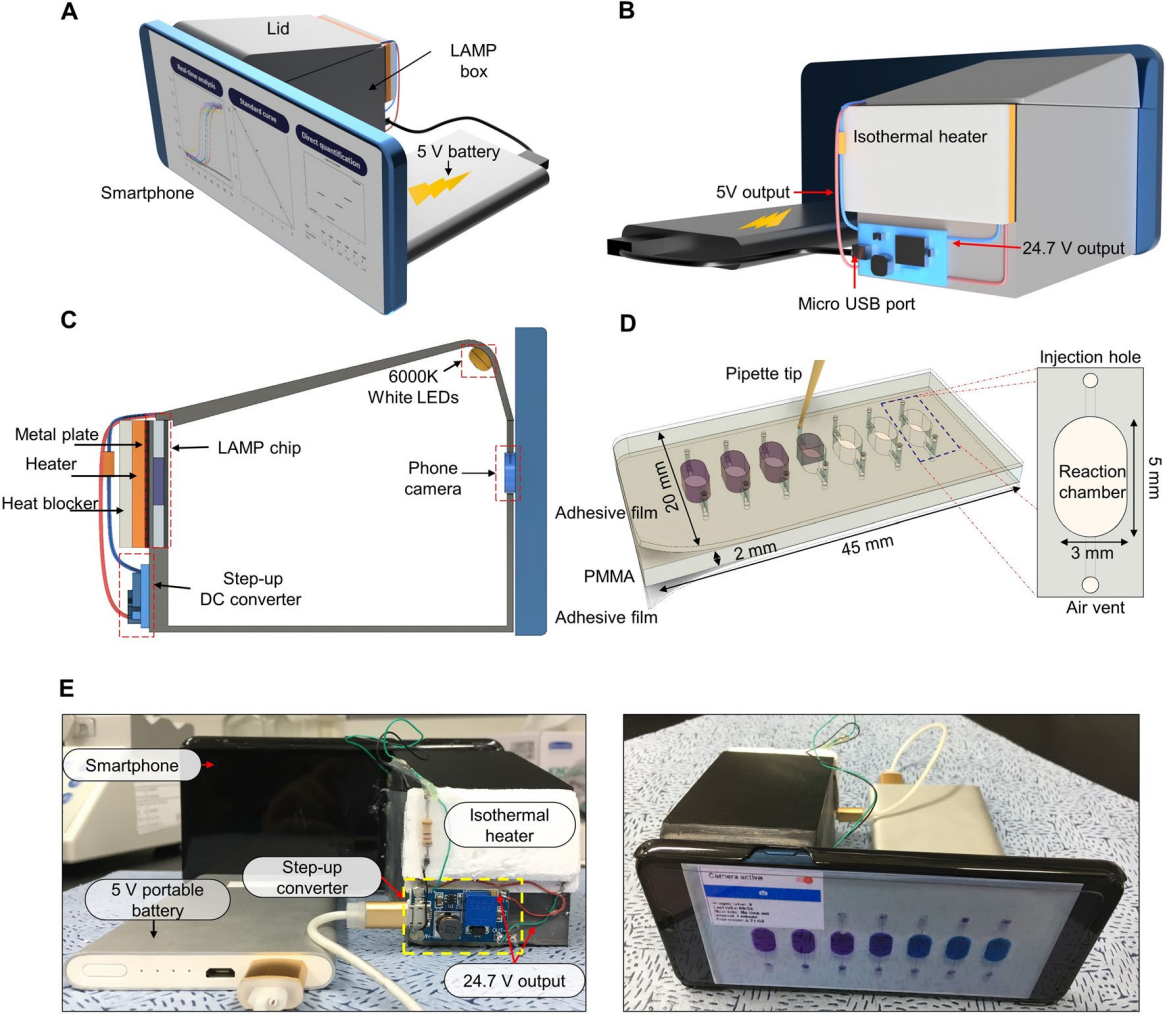
The schematics of the i-Genbox. The 3D models of the device are made using Fusion 360 (Autodesk, USA). (**A**) A tilted view of the i-Genbox. (**B**) A backside view of the i-Genbox, which consists of a commercial smartphone, a LAMP box, and an auxiliary battery. (**C**) A side view of the i-Genbox. On the left side of the LAMP box, there are a LAMP chip, a metal plate, an isothermal film heater, a heat blocker, and a step-up DC converter. On the right side, there is a smartphone camera in alignment with the reaction chamber of the LAMP chip. On the top side, a white LED array is installed. (**D**) The LAMP chip is designed with 7 chambers that are covered by an adhesive film in both sides. (**E**) The real images of the front side and the backside of the i-Genbox.

For the quantitative as well as qualitative analysis of the pathogens using the colorimetric LAMP reaction, we developed an integrated gene box (i-Genbox), which consists of a commercial smartphone, an auxiliary USB battery (5 V), a LAMP reaction box (6 cm height × 5 cm width × 8 cm length) equipped with a white LED, a LAMP chip, a DC converter, a film heater (Fig. 1A,B). Figure 1C shows the side view of the i-Genbox. The smartphone camera is positioned opposite to the LAMP chip, and five white LEDs (6,000 K) are installed on the top to shine a light toward the LAMP chip. The CMOS camera sensor of the smartphone serves as a detector by capturing the image of the reaction chamber every 1 min and as a data analyzer by processing the acquired images in the mobile app. On the backside of the chip, there are a metal plate and a film heater. The USB battery acts as a DC power supplier for the LEDs via a 50 Ω resistor as well as for isothermal heater via a step-up DC converter, which increases the input 5–24.7 V. At a voltage of 24.7 V, the temperature of the film heater was maintained at 65 °C that is adequate for LAMP reaction. The disposable LAMP chip has seven reaction chambers with an oval shape (5 mm height × 3 mm width × 2 mm depth) (Fig. 1D). The real image of the i-Genbox is shown in Fig. 1E. The LAMP box was fabricated by a 3D printer, and the components such as white LEDs, a film heater, a DC converter, and a LAMP chip were installed inside the LAMP box. And then, the camera window of the smartphone was positioned, and the USB battery supplied power to the DC converter for heating and lighting via a micro USB port.

### Strategy of the colorimetric analysis

The back-illuminated Exmor R CMOS image sensor of a smartphone camera technically writes the images in three color channels (red (R), green (G), and blue (B)) with a maximal sensitivity at 459 nm (B), 520 nm (G) and 597 nm (R)^11^. Thus, a customized color model can predict the amplification process, so the real-time monitoring of the colorimetric LAMP reaction can be executed quantitatively as well as qualitatively using a smartphone. First, it is important how to define the image area in multiple reaction chambers for colorimetric analysis. For this purpose, we developed an auto-select algorithm, which can distinguish reaction chambers from the background and eliminate air bubbles produced inside the chamber during the LAMP reaction (Fig. S1A). In the auto-select algorithm, there are three parameters (Threshold, Shrink, and Grow) to recognize the chambers and bubbles. For example, Fig. S1B shows the auto-select area by tuning the Shrink value as 1, 5, and 10, and the Shrink value of 10 clearly excluded the bubbles inside the chamber. The use of the auto-select algorithm enables us to perform the colorimetric analysis for the chamber with high consistency and is better than the fixed layout that includes the whole seven chambers, lessening the errors in the reading of color signals. After applying the auto-select mode of the reaction chambers, the average intensities of R, G, and B were computed and recorded for each chamber. Then, the average intensities of R, G, and B values were used to calculate the ratio of G/R and B/R. Another color indicator was the hue value. Normally, the hue values are coordinated in degree from 0° to 360°. However, for the purpose of comparing and calculating with/from RGB values, we normalized the hue values in the range of 0 to 1 instead of 0°–360° by dividing 360. Thus, the normalized hue value was obtained as follows: if R is max, then hue = 1/6 × (G − B)/(R_max _– R_min_); if G is max, then hue = 1/3 + 1/6 × (B − R)/(G_max _− G_min_); if B is max, then hue = 2/3 + 1/6(R − G)/(B_max _− B_min_), where the range of the RGB value is from 0 to 1. Hue value is an attribute of HSV (hue, saturation, value) color space which is the alternative representations of the RGB color model, defined technically as the degree to which a stimulus can be described as similar to or different from stimuli that are described as red, green, blue, and yellow^12^. In the HSV model, the hue value shows the color property itself regardless of the brightness or chromatic intensity, which theoretically makes the hue the suitable parameter to distinguish the color of an object under various lighting conditions.

**Figure 2:**
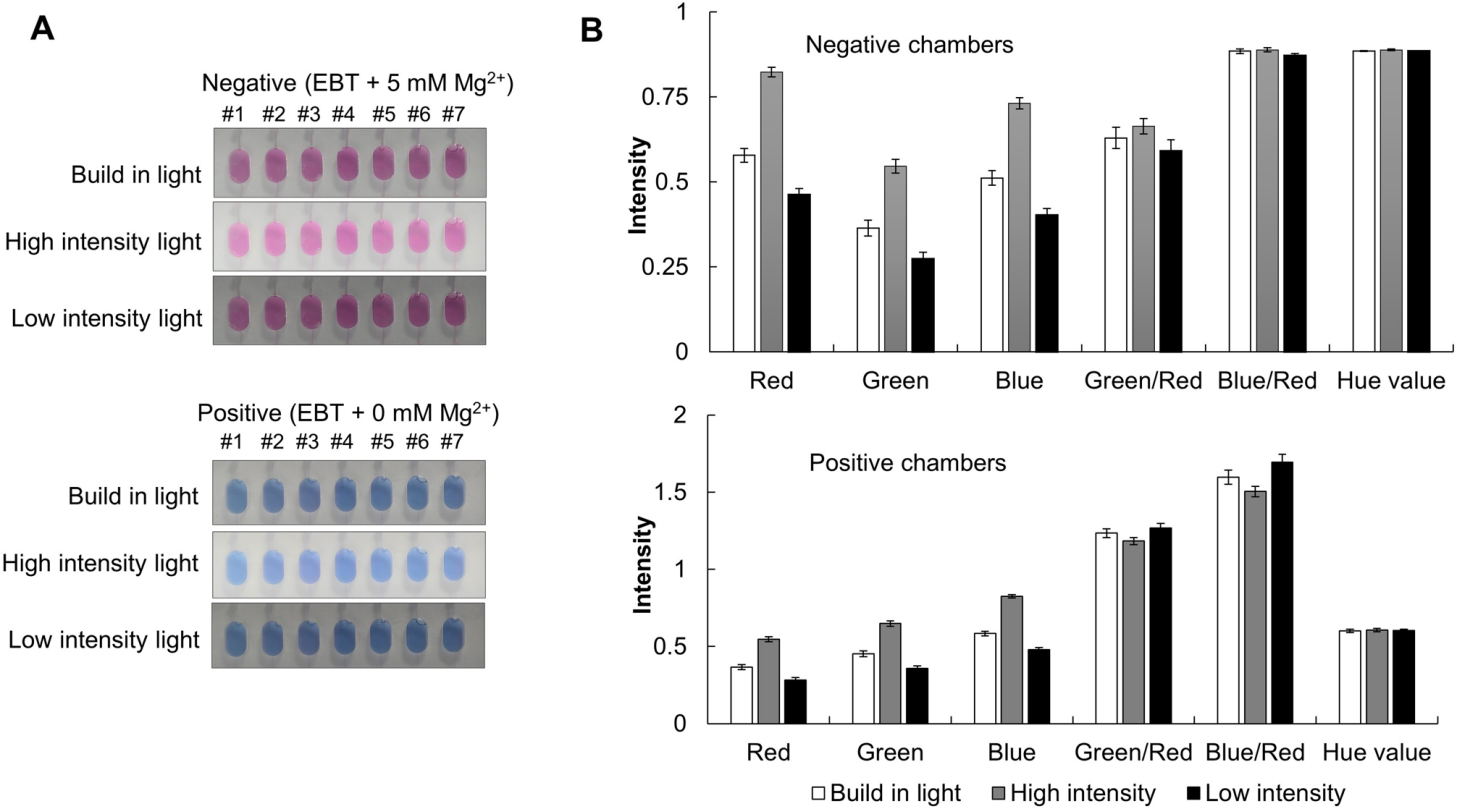
Comparison of the various colorimetric values for the negative and positive control depending on the light intensities. (**A**) Chip images of a negative control and chip images of a positive control, which were taken under the built-in light, high-intensity light, and low-intensity light. (**B**) The intensity values of various colorimetric analysis for the negative control and the positive control. The standard deviation (SD) was calculated from the values of seven chambers on the chip.

Since the colorimetric analysis approach uses a light source to visualize the color of the solution, the light intensity is an important factor and has a huge impact on the color uniformity. When the light intensity is low, the degree of color tone and homogeneity may suffer from the light source variation and the fluctuation of light intensity during the LAMP reaction. Therefore, it is necessary to assess the effect of the light source itself to guarantee the consistent results. We prepared for two solutions (a negative control: purple, 3 mM EBT + 5 mM Mg^2+^ and a positive control: blue, 3 mM EBT + 0 mM Mg^2+^), and a variety of colorimetric analysis methods were performed depending on the intensity of the light sources (a built-in light using LEDs (5 W) as installed in Fig. 1C, a high-intensity light using an external lamp (25 W, 20 cm away from the chip), and a low-intensity light using a room light (25 W, 1.5 m away from the chip). Figure 2A show the real digital images of the chip. Because the previous colorimetric analysis approaches used the absolute intensity of a single color or the ratio-metric intensity of the two colors to judge the nucleic acid amplification^7,13^, we evaluated the color intensity of each reaction chamber by R, G, B, G/R, B/R, and hue value (Fig. 2B). Except the hue value, the absolute color intensities of a single color (R, G, B) and the ratio-metric intensities of the two colors (G/R and B/R) show huge variation depending on the light source. In addition, the differences of the color intensity from chamber to chamber are also significant (Fig S2). On the other hand, the hue value revealed its consistency regardless of the kind of the light source and the chamber position, demonstrating the robustness of the hue value as the colorimetric analysis tool (Fig. 2B).

**Figure 3:**
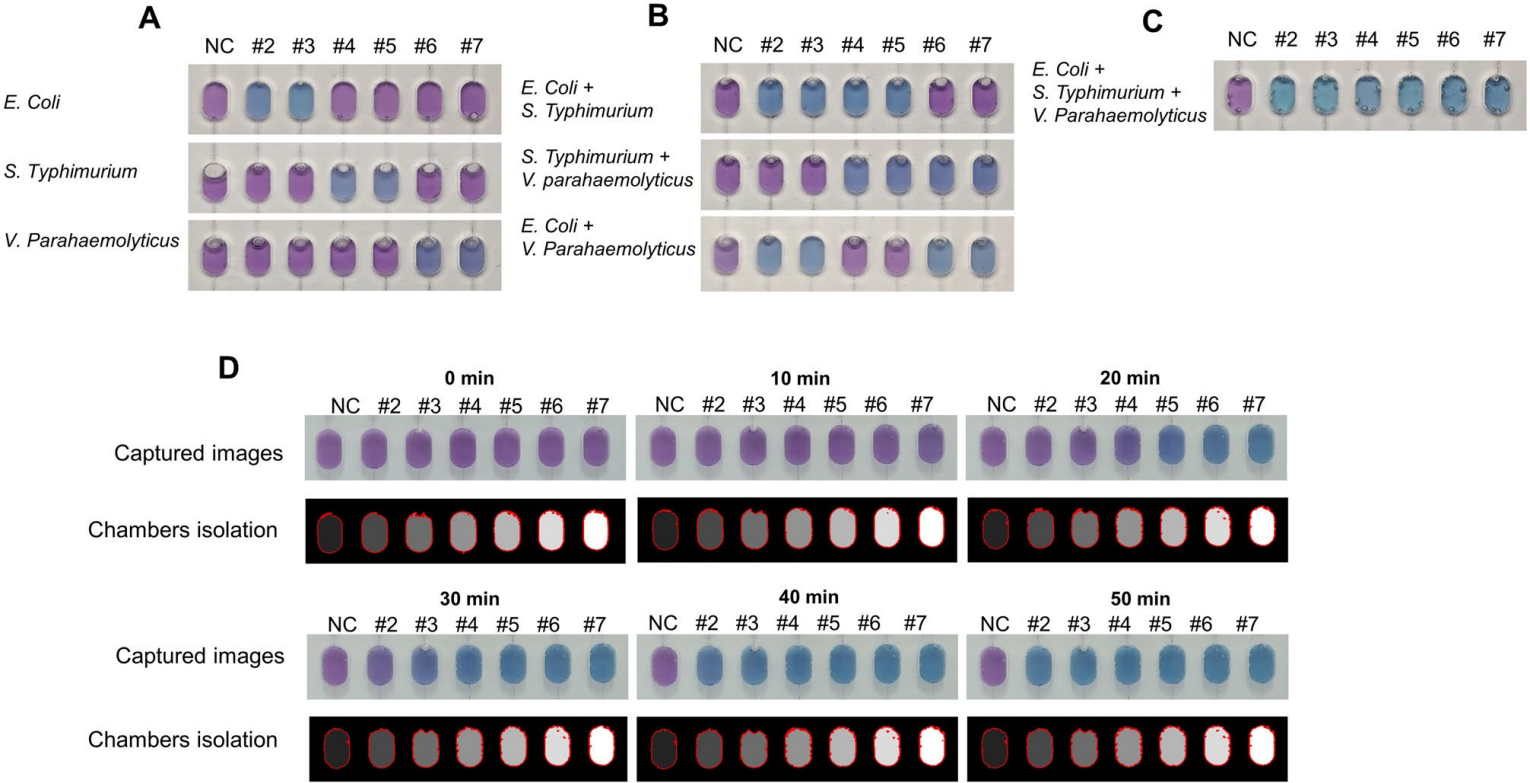
Colorimetric qualitative and quantitative of LAMP assay. (**A**) Monoplex, (**B**) duplex and (**C**) triplex bacteria identification can be performed with ease by monitoring the color change at the endpoint. (**D**) Quantitative analysis of the pathogenic bacteria on the i-Genbox. Time-lapse images were acquired every 1 min and some representative images (every 10 min of the reaction time) are shown. The input amount of the genomic *E. coli* DNA serially increased from the NC chamber to the chamber #7, thereby starting the color change from the chamber #7 to the chamber #2. For the colorimetric analysis of each chamber, the auto-select mode was employed for automatic definition of the reaction chamber and the removal of air bubbles, which is marked by a red boundary. The grayscale intensity in the chambers accounts for the chamber number, meaning the darkest gray chamber # 1 and the brightest gray chamber # 7.

As a first step, we executed the qualitative genetic analysis using three pathogenic bacteria (*E. coli W*, *S. Typhimurium*, and *V. parahaemolyticus*). The primer sets for amplifying the target genes (*FliC* of *E. coli W*, *invA* of *S. Typhimurium,* and *ropD* of *V. parahaemolyticus*) were pre-coated in the chamber #2&#3, #4&#5, and #6&#7, respectively, while the first chamber served as a negative control (NC) in which no primers were loaded. The genomic template of each bacteria was prepared, and the monoplex, duplex, and triplex bacteria detection was conducted. Figure 3A shows the colorimetric images of the LAMP chip. In the monoplex test, only the chamber, which contained a primer set to match with the designated bacterial template, underwent the color change from violet to blue. In a same way, the duplex bacterial identification was completed with success. For example, the chamber #2&#3 and #5&#7 shows the color change when the genomic templates of *E. coli* W and V. parahaemolyticus were injected (Fig. 3B). When the genomic templates for the three bacteria were loaded together, the color of all the chambers changed from violet to blue (Fig. 3C). These results mean that the primer set is specific for each target, and the contamination issue does not occur during the LAMP reaction on a chip. The multiplexing capability can be expanded simply by increasing the number of the reaction chambers with ease.

For the quantitative analysis of the colorimetric LAMP reaction in the i-Genbox platform, we chose *E. coli W* as a model, and loaded serially diluted genomic templates into the chamber: 1.32 × 10^2^ copies for the chamber #2, 1.32 × 10^3^ copies for the chamber #3, 1.32 × 10^4^ copies for the chamber #4, 1.32 × 10^5^ copies for the chamber #5, 1.32 × 10^6^ copies for the chamber #6, 1.32 × 10^7^ copies for the chamber #7. When 10^1^ copies per the chamber was used, the success rate was ~ 60%, so the limit-of-detection was 10^2^ per chamber. Figure 3D shows the digital time-lapse images and the auto-select images to display the progress of the color change of each chamber. On-chip images were recorded every 1 min by a smartphone camera using the i-Genbox (Supplementary Video 1). The representative pictures show that the color change was initiated at 10 min for the chamber #7, and after 20 min, the color of the chamber #5 and #6 was also changed to blue. When the reaction proceeded for 30 min, the color of the chamber #2 was clearly turned, and all the chambers revealed blue after 40 min.

**Figure. 4:**
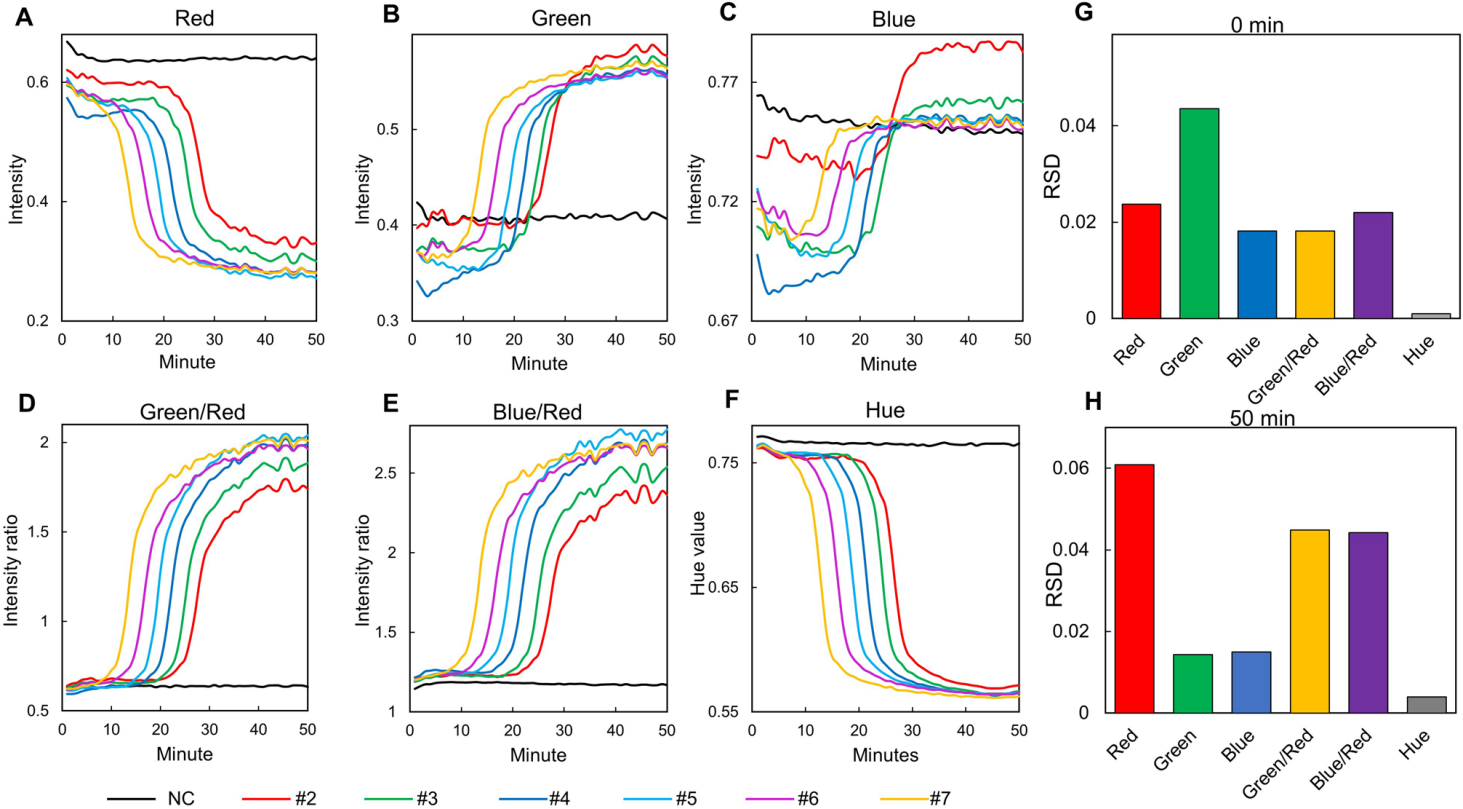
Screening of various colorimetric approaches for real-time LAMP reactions. (**A**–**C**) R, G, and B intensity profiles in the LAMP chambers along with the reaction time. (**D**,**E**) The ratio-metric profiles of G/R and B/R intensities along with the reaction time. (**F**) The hue values showed great correlation between the reaction time and the DNA copy number, and the hue values at the beginning stage and the final stage were very equivalent among the chambers. (**G**,**H**) The RSD shows the variation of the color value in the six reaction chambers before and after the LAMP reaction depending on the colorimetric analysis method. The hue approach displayed the least variation among the reaction chambers at the reaction time of 0 min and 50 min.

To analysis multiple reaction chambers at once, the best colorimetric approach should show less variation of the color measurement values among the chambers at the initial state (the first captured images in Fig. 3D) as well as the chambers at the end of the LAMP reaction (the last captured images in Fig. 3D). Using the time-lapse images of the LAMP reaction, the change of the individual R, G, and B intensity was extracted and plotted against the reaction time (Fig. 4A–C). Figure 4G and H shows the relative standard deviation (RSD) of each colorimetric approach. Although color intensities have varied during the amplification with high correlation to each other (Fig. S3), the difference of the color values among the chambers are noticeable at the first and the last measurements (Fig. 4G,H). The ratio-metric, G/R and B/R also displayed these fluctuations (Fig. 4D,E). These ratio-metric approaches showed less dispersion of the colorimetric values among the chambers at the beginning of the reaction, but the huge dispersions appeared at the end of the reaction (Fig. 4G,H). However, the hue value revealed the least variation of the colorimetric values among the chambers before and after the LAMP assay and the excellent LAMP profiles in accordance with the genomic DNA copy number, demonstrating the most appropriate approach for real-time monitoring of LAMP (Fig. 4F–H). Therefore, the hue value is the best choice to monitor real-time LAMP reactions, because it produces the lowest variation for the colorimetric analysis regardless of the chamber position and shows the consistency regardless of the light sources (Fig. 2).

### Quantitative analysis of the real-time colorimetric LAMP reaction

**Figure 5:**
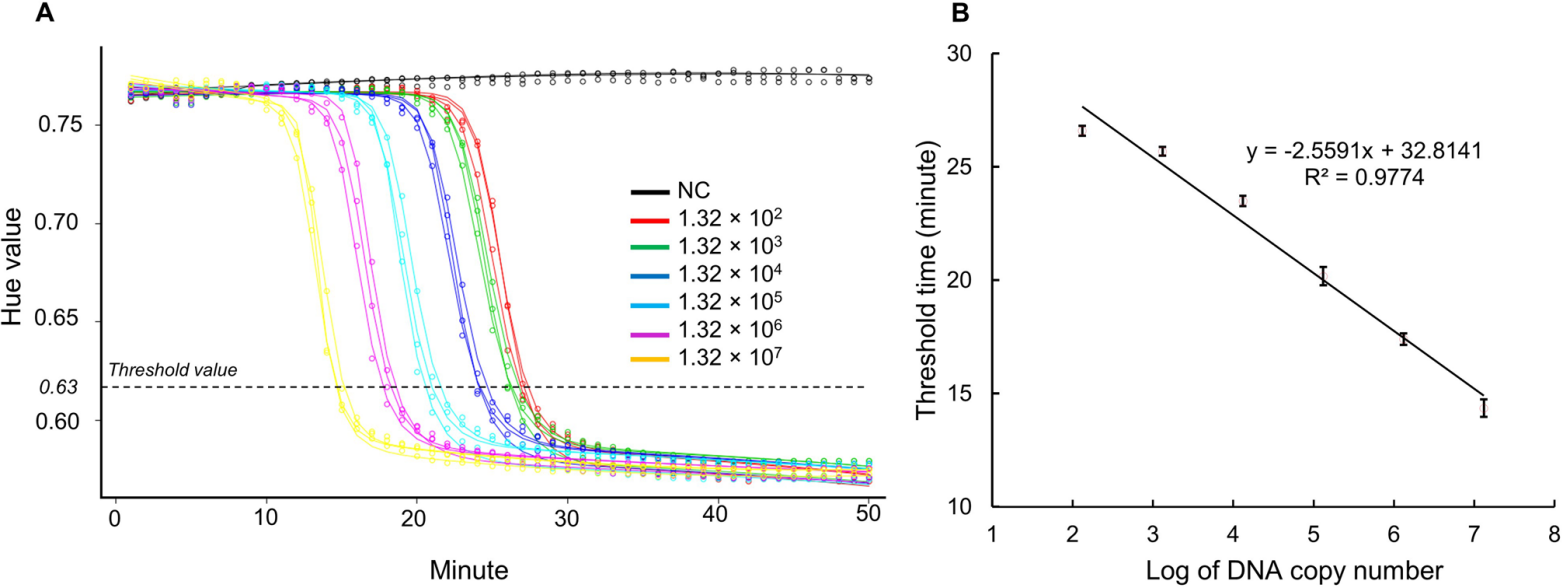
Quantitative analysis of the colorimetric qLAMP reaction. (**A**) Thrice experiments were performed with the LAMP chip, which contained the *E. coli* genomic DNA. The color of each chamber was recorded in the i-Genbox every 1 min, and the image data were analyzed by the hue value on the smartphone. (**B**) As the threshold value was set as 0.63, which was determined by the second derivative method, the threshold time was obtained for each chamber. By correlating the threshold time with the logarithm of the DNA copy number, the calibration curve can be generated for the quantitative analysis.

Sigmoidal model fitting is one of the established methods for the analysis of qPCR data to create a non-linear or logistic regression from fluorescent signals. Then, the threshold cycle (C_t_) can be predicted with a threshold value to obtain the quantitative information from the calibration curve^14–16^. In this study, the hue values obtained from thrice experiments were offered to create 4, 5, 6, and 7-parameter sigmoidal fitting curves. The Akaike information criterion (AIC) and AIC weight were used to estimate the relative quality of these statistical models with the hue values^17,18^. The seven-parameter sigmoidal model shows the lowest AIC score and the closest to 1 of AIC weight in 14 out of 18 datasets (Table S1 and S2). Thus, we selected the 7-parameter sigmoidal model for the further experiments. Details of the fitting methods used in this study was presented by Spiess et al.^17^. To produce the quantitative data of E. *coli W*, the LAMP reaction was carried out with a serially diluted copy number of genomic DNA templates as described in Fig. 3D. The time-lapse images were obtained at the interval of 1 min, and the hue values of the reaction chambers were fitted using the 7-parameter sigmoidal model. Figure 5 shows the amplification profiles of each chamber, and the curve behaviors were different depending on the concentration of genomic DNA. The curve of the chamber containing 1.32 × 10^2^ DNA copy number (red) has fallen later than the curve of the chamber containing 1.32 × 10^3^ DNA copy number (green). In a similar way, the curve of the chamber containing the highest DNA copy number (yellow), 1.32 × 10^7^, has fallen in the first. Since the color of the LAMP cocktail changed from violet to blue, the qLAMP profile started at a high value of the hue, and then went down as the reaction time proceeded, which seems upside-down to the conventional qPCR. The threshold value (~ 0.63) was set using the second derivative maximum method as qPCR^19,20^, and accordingly the threshold time was obtained from the profiles of Fig. 5A. By correlating the threshold time and the logarithm of DNA templates, a linear regression plot was conducted, producing the standard quantitative curve (R^2^ = 0.9774) in Fig. 5B.

### Direct quantification using a mobile app on the smartphone

When the best colorimetric method is established, we developed a mobile web-based app for colorimetric analysis using an R environment and shiny package, named “qLAMP app”. Since the web-based app can be easily deployed in different types of smartphones running Android and iOS, the popular computing platforms such as Window, macOS and ChromeOS devices can access the qLAMP app. The developed mobile app executes the following procedure in a sequence: isolation of the reaction chamber by the auto-select mode, calculation and plotting of the hue value for the color transition in each chamber throughout the reaction time, calculation of the threshold value, and determination of the threshold time for each chamber. To do this, the qLAMP app was designed with two modules, which can be accessed via https://inanobiomems.shinyapps.io/qLAMP. The first module, “Image Processing”, is responsible for the first two steps, and the second module, “Data Processing”, is for the remaining steps. The first step is to go to the image processing tab, where we can upload all the image files (for example, 50 files in this case). In order to reduce the data processing time and data transfer between the server and the smartphone, the captured images were cropped to a smaller size before uploaded (Fig. S4). Then, adjust auto-select parameters including Threshold, Shrink, and Grow to execute the auto-select in order to distinguish the chamber area from the background. In our case, the optimal values for Threshold, Shrink, and Grow were 29, 1, and 1. We can see both the uploaded images and the auto-select images in parallel in the bottom. Once the auto-select mode is applied, the hue values are computed at the same time. On the bottom, there is a table to show the hue value for each chamber for every minute. Next, the data processing tab should be clicked. In this tab, we can see two modes of analysis: Standard Calibration Curve and Quantification of Real Sample. First, we need to produce the standard calibration curve, so we click the Standard Calibration Curve mode. To obtain the standard curve, we carried out the same experiments as shown in Fig. 5 and uploaded 50 image files that were taken every minute during the LAMP reaction. Then, we added the value of 1.32 × 10^2^ in the box of the Lowest Concentration, and 10 in the box of the Dilution factor. Under those conditions, the mobile phone automatically fits the hue value into the 7-parameter sigmoidal model, producing the amplification profiles of qLAMP, which is shown as the first graph. Simultaneously, it calculates the second derivative maximum of the whole data, which is used as the threshold value. In our case, the threshold value was determined as 0.63. Since the threshold value was set as 0.63, the threshold time can be accordingly decided for each diluted sample from the amplification profiles. Finally, the standard calibration curve displaying the relationship between the threshold time and the logarithm of DNA copy number is generated as the second graph. The standard curve is expressed as a linear regression line. The circle markers represent six data points derived from the serially diluted DNA samples. On the bottom, there is a table to show the digital numbers of the threshold time versus the DNA copy number. To save data, click the ‘Save as Calibration Curve’ button at the end of the page, which will produce a file for the standard curve (qLAMP.csv) in the Download folder and used for the quantification of real samples later (Supplementary Video 2).

For the quantification of real samples, the first chamber of the LAMP chip should be a negative control, while the chamber #2 to #7 contains unknown DNA copy number. After completing the qLAMP reaction, we undergo the same steps of ‘Imaging Processing’ as described above. Then, we move to the ‘Data Processing’ tab and choose the mode of ‘Quantification of Real Sample’. The values of Lowest Concentration and Dilution Factor are set as 1.32 × 10^2^ and 10, respectively. Next, we upload the standard calibration curve by browsing qLAMP.csv. This process will display the amplification profiles of the reaction chamber containing real samples as well as six square markers of each sample in the standard calibration curve. Finally, there is a table showing the data of the logarithm DNA copy number for each reaction chamber (Supplementary Video 3).

**Figure 6:**
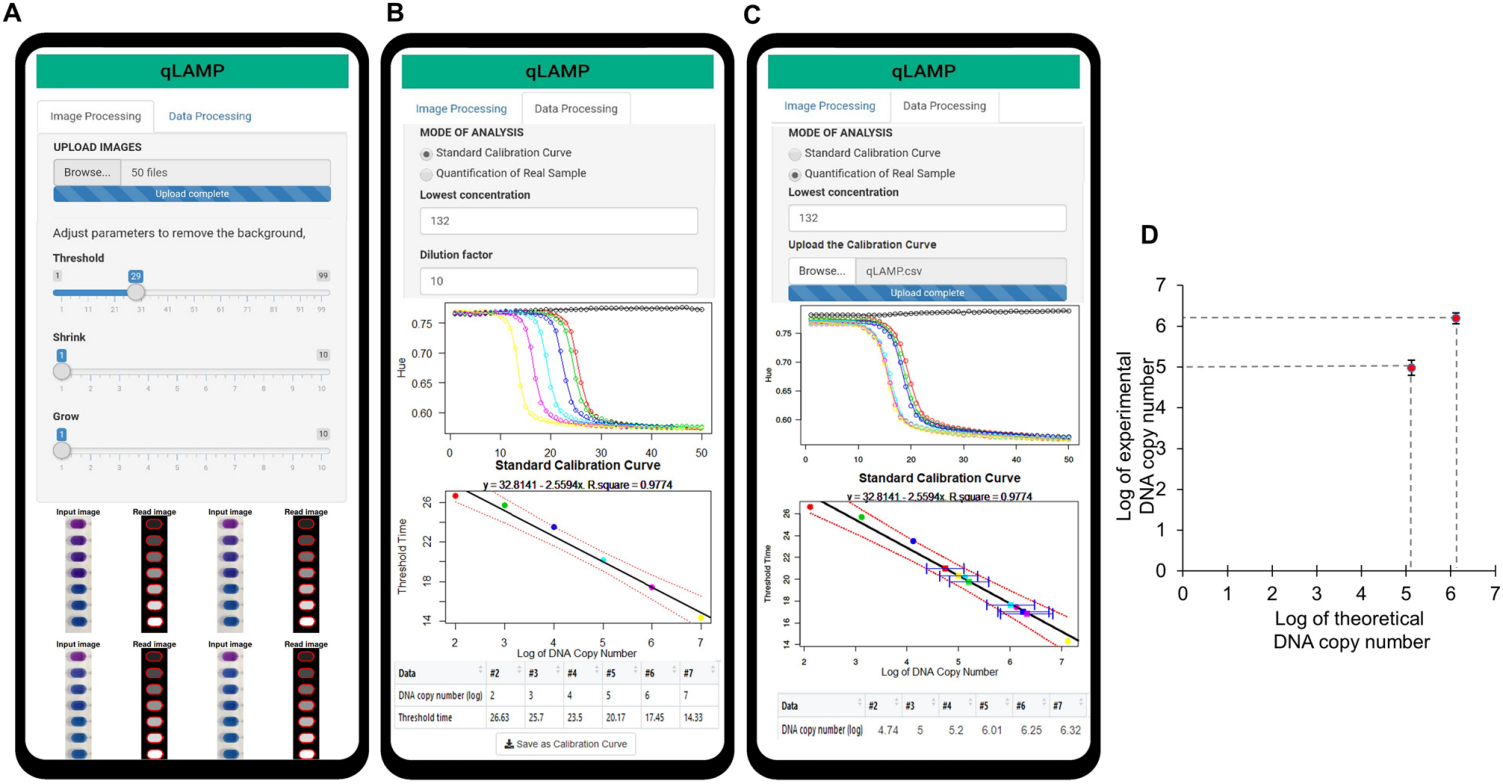
Direct quantification of *E. coli* genomic DNA. (**A**) the cropped images are uploaded and then the auto-select mode is applied. The color value is extracted from the reaction chamber and the hue value is computed in the mobile qLAMP app. (**B**) With the mode of ‘Standard Calibration Curve’, the serially diluted samples generated respective qLAMP profiles and the threshold value which is determined by the second derivative maximum revealed the threshold time for each diluted sample. Thus, the standard curve plotting the threshold time versus the logarithm of DNA copy number can be generated, the black line represents the linear fit to the data, the red dotted lines indicate the 95% confidence interval. (**C**) With the “Quantification of Real Sample” mode, the qLAMP profile of the real sample is produced, and the threshold time is decided by the threshold value. The threshold time is marked in the calibration curve, showing the DNA copy number in the real sample. (**D**) The high correlation between the theoretical DNA copy number and the experimental DNA copy number is shown. Figure (**A**–**C**) are the screenshots from the smartphone during the analysis.

To demonstrate the capability of the proposed qLAMP app, we performed an additional LAMP assay, in which the LAMP reaction proceeded at 65 °C for 50 min on a chip that contained one chamber without DNA templates, three chambers from #2 to #4 with 1.32 × 10^5^ copy numbers, and other three chambers from #5 to #7 with 1.32 × 10^6^ copy number. The color image of the chambers was taken every 1 min. Following the ‘Imaging Processing’ mode, we could obtain the hue value for each chamber at each time (Fig. 6A). We used the same calibration curve as above (Fig. 6B) and uploaded it for quantifying the real samples. The mode of ‘Quantification of Real Sample’ produced the qLAMP profiles, the square markers in the calibration curve, and the data table (Fig. 6C). The threshold time of the chamber containing 1.32 × 10^5^ DNA copy number was determined as approximately 17 min, which was calculated to 5.00 of the logarithm of DNA copy number (SD = 0.188) from the standard calibration curve. Theoretically, the logarithm of 1.32 × 10^5^ copy number is 5.12 in the standard calibration curve. Thus, the theoretical value (5.12) is quite similar to the experimental value (5.00) (Fig. 6D). In a same way, the threshold time in the qLAMP profile for the chamber containing 1.32 × 10^6^ copy number was determined as approximately 20 min, which corresponded to 6.19 of the logarithm of DNA copy number (SD = 0.133). The theoretical value of the logarithm of 1.32 × 10^6^ copy number is 6.12. Therefore, the experimental quantitative values were quite matched with the theoretical values, showing the validity and fidelity of our proposed qLAMP app for DNA quantification.

## Discussion

In the conventional qPCR, the amplification process is time-synchronized and dependent on the thermal cycling number, and each cycle should provide enough time for DNA duplication. With tenfold serially diluted samples, the ideal slope of the standard curve for qPCR is -3.32, indicating that the efficiency of the amplification is 100% per cycle. The amplification efficiency can be calculated by the following equation: Amplification efficiency = (10^(−1/slope)^—1) × 100. On the other hand, the LAMP reactions take place in parallel at constant temperature, so the reaction time instead of the reaction cycle is measured for characterizing the amplification process. Our results for the quantitative calibration curve of *E. coli* W show a correlation between the logarithm of DNA copy number and the threshold time with the slope value of − 2.56 (R^2^ = 0.9774) (Fig. 5B), which means that the amplification efficiency is 145.82% per min. The amplification efficiency above 100% implies that the reaction speed of the LAMP is much faster than the qPCR for duplication. In fact, the reaction time of LAMP is normally completed less than 1 h, while qPCR takes more than 2 h. The colorimetric assay enables us to qualitatively judge the success of the rapid LAMP reaction with ease, but the quantitative analysis for the colorimetric LAMP has been considered difficult due to vague definition during the color transition and lack of digital description for color change. However, the recent development of the sensitive CMOS sensor on smartphone allows us to accurately obtain the individual value of R, G, and B of the color, so we can digitally monitor the color transition of the colorimetric LAMP reaction anytime anywhere. Using the hue value, the amplification profile shows the reverse pattern compared with qPCR that starts from higher hue value to lower one, as the color is changed from violet to blue. Once the threshold hue value is set, the threshold time is automatically generated for each sample. Thus, the relationship between the DNA copy number and the threshold time can be produced, and we can use it for quantitative analysis of the sample.

Even though the colorimetric method is cost-effective and simple compared with a fluorescent dye, the color tone of the LAMP reaction could be changed depending on the environments such as brightness and direction of light. To address this issue, we demonstrated that the use of the selected colorimetric approach with the hue value was robust and consistent regardless of lighting conditions and chamber positions. The auto-select algorithm significantly improves the accuracy of data analysis by distinguishing the color of the multiple reaction chambers from the background and removing bubbles formed inside the reaction chambers. We also introduced an open-source analytical tool with a web-based interface, which can be easily accessible using a smartphone. It allows us to perform quantitative analysis of genomic copy number simply by observing the color transition on the smartphone CMOS camera. The customized mobile app utilized the computing ability of the smartphone for color measurement, data analysis, and data storage.

We demonstrated the feasibility of the quantification of colorimetric isothermal amplification on the smartphone and its open-source app using the purified genomic DNA templates as well as the bacterial cells (Fig. S6). In case of the bacterial cells, we used the direct LAMP reaction by adding EZ-way buffer in the LAMP cocktail so that the cell lysis and LAMP reaction could be carried out serially. Compared with the purified DNA templates, the direct LAMP reaction with the EZ-way buffer took longer, but the relationship between the threshold time and the bacterial cell number was well correlated.

In summary, we demonstrated that the colorimetric assay could be used for a real-time quantification of nucleic acid. We developed a methodology for a smartphone-based system for quantitative analysis of nucleic acid in multiple reaction chamber at same time by selecting a suitable colorimetric color dye, choosing an appropriate colorimetric approach, and establishing a workflow for quantitative analysis of color change, and setting up the relationship between the threshold time and the amount of DNA. Our platform can be applied to any universal smartphone for the quantitative and qualitative POCT DNA testing, especially in a low- resource setting.

## Methods

### Fabrication of the i-Genbox and the LAMP chip

The LAMP box was designed with a 360 Fusion software (Autodesk, USA), and manufactured in black by a Cubicon DLP 3D 110D printer (Cubicon, Korea) (Fig. 1). The designed .stl file can be found in the website for the qLAMP app. The isothermal heater film, Minco SmartHeat, was purchased from Minco (USA) and cut into a small piece (3 cm × 5 cm). The SmartHeat heaters utilize an exclusive polymer inner-lay material to pinpoint precisely when and where heat is needed to maintain uniform heatsink temperature. The resistance of the polymer increases exponentially at a prescribed temperature control point, which eliminates the need for additional regulating electronics. In other words, the temperature of the heater can be controlled and maintained at a constant temperature simply by applied a stable input voltage. When the voltage was set as 24 V, the temperature of the heater is maintained constantly at 60 °C. Experimentally, we found that the temperature of 65 °C was achieved with 24.7 V, which was used for the LAMP reaction. The step-up DC converter (MT3608, Gousheng, China) was used to boost 5 V of an auxiliary battery to 24.7 V.

The LAMP chip was designed with a Cut2D program (Vectric Ltd, USA) and was manufactured in a 2.0 mm-thick poly(methylmethacrylate) (PMMA) plate (Acrytal, Korea) using a CNC machine (Tinyrobo, Korea). The PMMA chip was sonicated in 70% alcohol to remove any dust and debris and was sterilized. An adhesive film layer (No. 900360, Hj-Bioanalytik GmbH, Germany) was used to cover both sides of the chip in a clean bench to prevent any contamination. A sterilized needle was used to punch the hole for the sample injection and the air vent holes in the chip. After loading the samples into the LAMP chamber, an additional layer of an adhesive film was attached to prevent the evaporation during the LAMP reaction.

### DNA preparation and primer design

The bacterial genomic DNAs were extracted from *E. coli W*, *S. Typhimurium*, and *V. parahaemolyticus* using a QIAamp DNA Mini kit (Qiagen, Germany) according to the manufacturer’s instructions. The *fliC* gene of *E. coli W, invA* of *S. Typhimurium*, and *ropD* of *V. parahaemolyticus* were targeted. A NanoDrop 3,000 (Thermo) was used to estimate the concentration of the DNA. The tenfold serially diluted DNAs were prepared with a TE buffer to obtain a calibration curve. The LAMP primer sets were designed in a PrimerExplorer V5 software. The sequence information of the forward and backward outer primers (F3 and B3), and the forward and backward inner primers (FIP and BIP) is shown in Table S3.

### Preparation of the LAMP reaction mixture

The LAMP reaction was carried out on chip chambers with a total volume of 30 µL. Each reaction chamber contains 2.55 µL of 10 × Isothermal Amplification buffer (New England Biolabs, USA), 1.5 µL of 100 mM MgSO_4_ (New England Biolabs, USA), 15.6 µL of 2.5 mM dNTPs (Takara Korea Biomedical Inc, Korea), 0.84 µL of 100 µM F3 primer, 0.84 µL of 100 µM B3 primer, 0.84 µL of 100 µM FIB primer, 0.84 µL of 100 µM BIP primer, 1 µL of 3 mM EBT (Sigma, Germany), 1 µL of a DNA sample, 1.24 µL of 5 M betaine (Sigma-Aldrich, USA) and 3.75 µL of 8000U Bst Polymerase 2.0 (New England Biolabs, USA). The LAMP box was powered for 10 min in advance for pre-heating the chip-holding site to approximately 65 °C. The LAMP reaction was completed at 65 °C for 50 min using the i-Genbox platform. The heating profile of the front side of the chip is shown in Fig. S5. For the qualitative experiments, the target primers were air-dried in designated chambers before sealing the chip.

### Colorimetric analysis

The whole process of the images analysis was done by using R environment (version 3.5.2). Required packages include shiny, shinyBS, imager, colocr, and qpcR. The auto-selection of the chambers was achieved by using a select (roi_select) function for the region of interest (ROI) from a package, named colocr^21^. In brief, it starts by selecting the chamber structure in the gray-scale image using the default values of three major operations; threshold, grow (dilation) and shrink (erosion). Threshold excludes the pixels below a certain value, which should be matched with the background. Grow and shrink make the boundary of the reaction chamber move outward and inward by several pixels, respectively, when we need to re-adjust the boundary. In this study, we set the value of the threshold, shrink and grow operations as 29, 1, and 1, respectively. The hue values were calculated by a built-in function of R (function name: rgb2hsv). The fitting models and the prediction of the DNA copy number were developed based on the qpcR package^22^. The AIC and AIC weight were calculated using the mselect function in qpcR package. The website interface was built by using a shiny package and hosted in a public address of https://inanobiomems.shinyapps.io/qLAMP/. The computer code and source code of the app used in this study is available at https://github.com/nqh2412/qLAMP/.

## Supplementary information

Supplementary Information

Supplementary Video 1

Supplementary Video 2

Supplementary Video 3

## Data Availability

All relevant data are included within the article and Supplementary Information files and available upon reasonable request from the authors.

## Code availability

The computer code and source code of the app used in this study is available at https://github.com/nqh2412/qLAMP/.
