# Supplementary material for: Quantification of colorimetric isothermal amplification on the smartphone and its open-source app for point-of-care pathogen detection
Source: Sci Rep. 2020 Sep 15;10:15123. doi: 10.1038/s41598-020-72095-3 (PMC7493899; doi:10.1038/s41598-020-72095-3)
Supplement: Supplementary file 1 — Supplementary Information [file 41598_2020_72095_MOESM1_ESM.docx]

**Supporting Information**

**Quantification of colorimetric isothermal amplification on the smartphone and its open-source app for point-of-care pathogen detection**

*Huynh Quoc Nguyen, Van Dan Nguyen, Hau Van Nguyen, Tae Seok Seo**

Department of Chemical Engineering, College of Engineering, Kyung Hee University, 1 Seochon-dong, Giheung-gu, Yongin-si, Gyeonggi-do 17140, Republic of Korea.

E-mail address: seots@khu.ac.kr; Phone: +82-31-201-3676; Fax: +82-31-204-8114


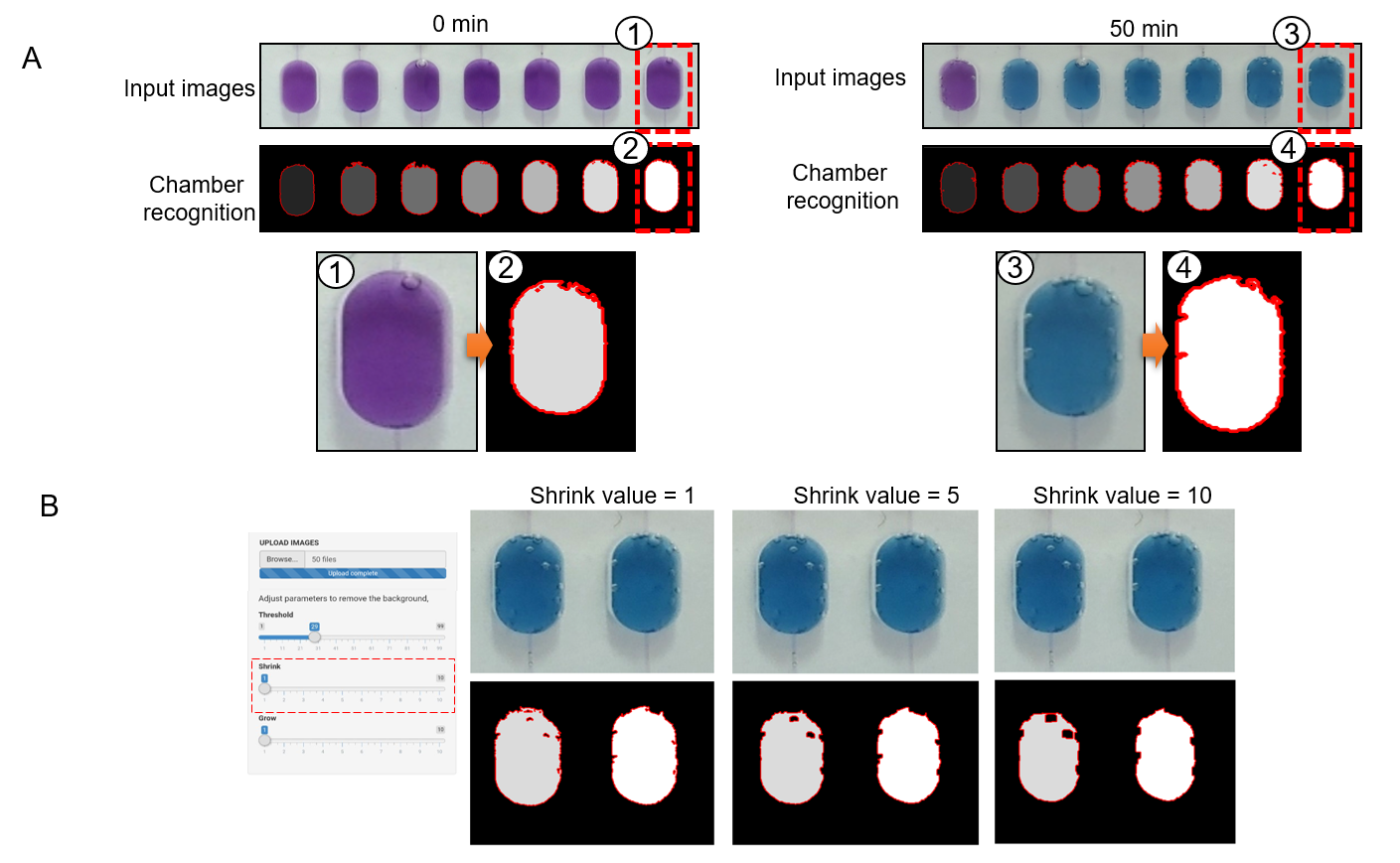


**Fig. S1**. (A) The auto-select mode was chosen as a chamber recognition algorithm to eliminate the air bubbles inside the reaction chamber. (B) The shrink value can be tuned in the app to clearly define the bubbles formed inside the chamber.


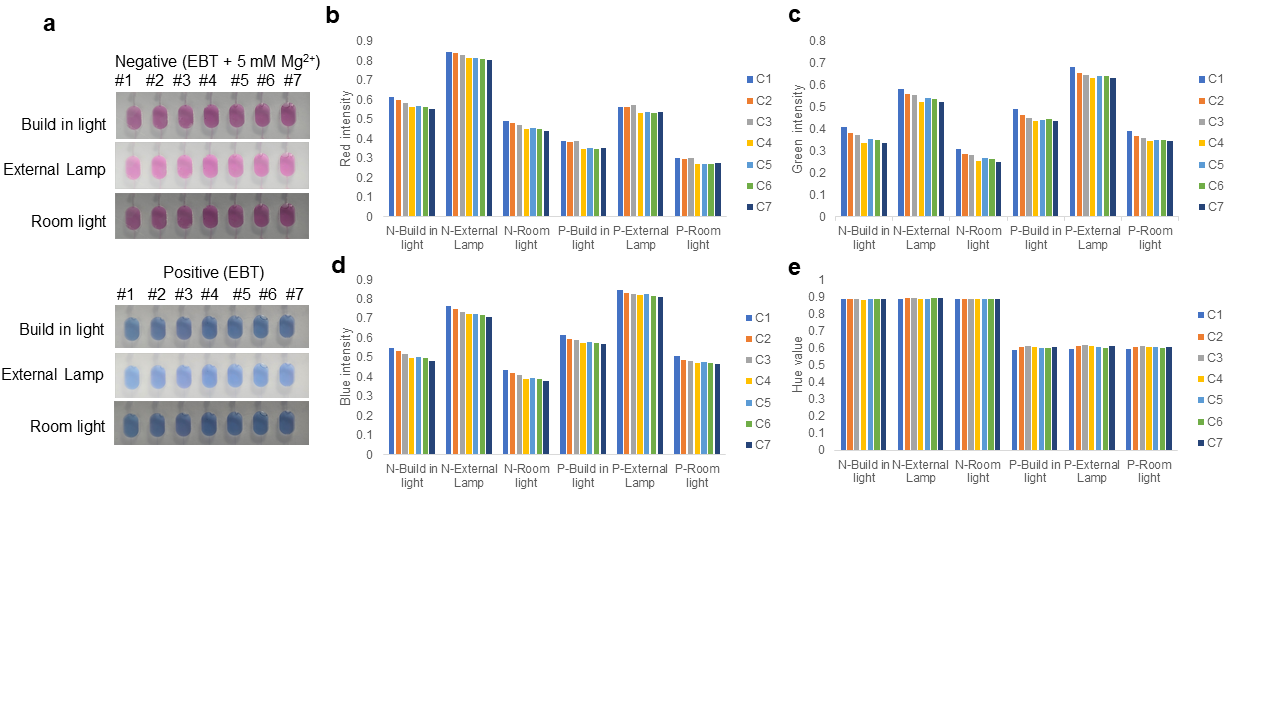
**Fig. S2**. Comparison of the colorimetric value of the negative (EBT + 5 mM Mg^2^) and positive (only EBT) control under various light conditions. (A) The Images of the LAMP chip, which were taken under a built-in light using an LED array (5 W) as installed in Fig. 1, a high-intensity light using an external lamp (25 W, 20 cm away from the chip), and a low-intensity light using a room light (25 W, 1.5 m away from chip). (B) (C) (D)The intensities of R, G, and B are significantly fluctuated depending on the lighting conditions, and even among the reaction chambers. e The intensity fluctuation of the Hue value is minimal and consistent regardless of the light sources and the chamber position.


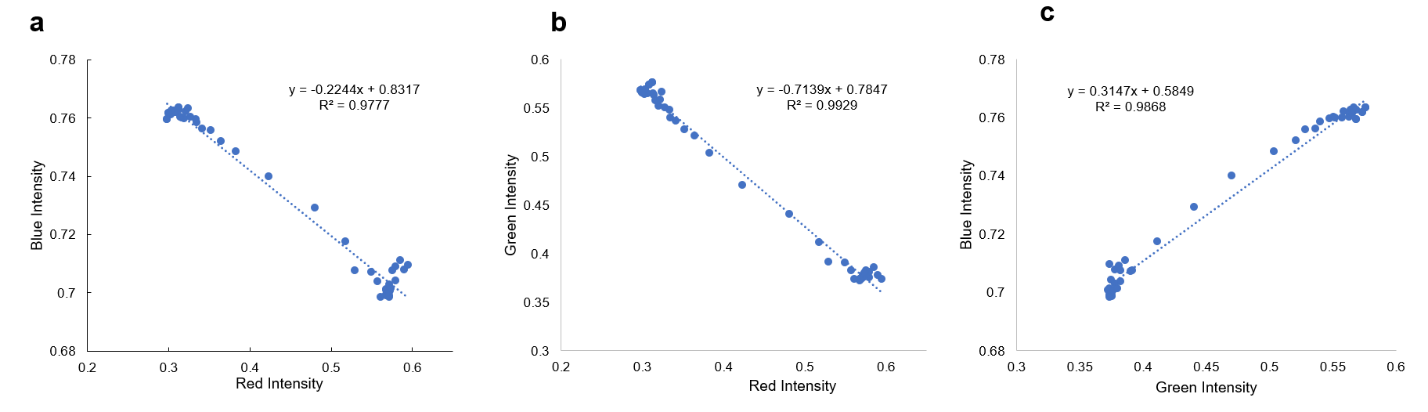
**Fig. S3**. Correlation of the color channels during the color transition in the LAMP reaction at the chamber #4. **(A)** Correlation between R intensity and B intensity. **(B)** Correlation between R intensity and G intensity. **(C)** Correlation between G intensity and B intensity. It suggests that all color intensities should be consider throughout the calculation.


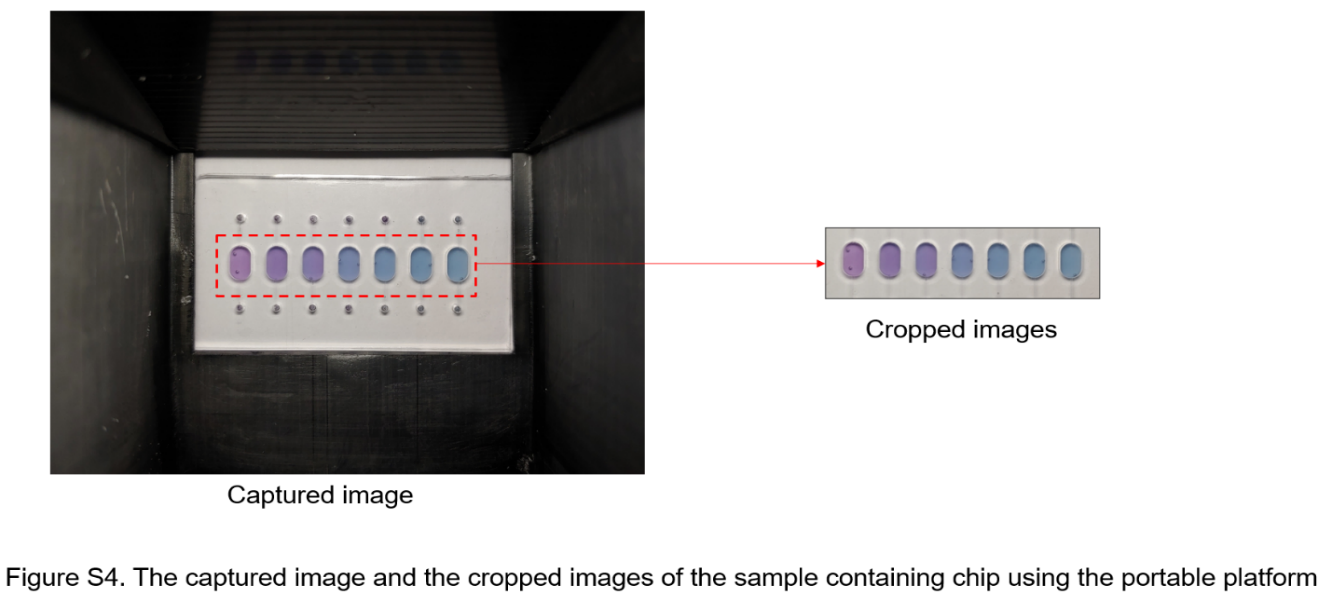


**Fig. S4**. To reduce the data transfer time between the smartphone and the web server, the captured image (the left image) is cropped to a smaller image, which is uploaded (the right image).


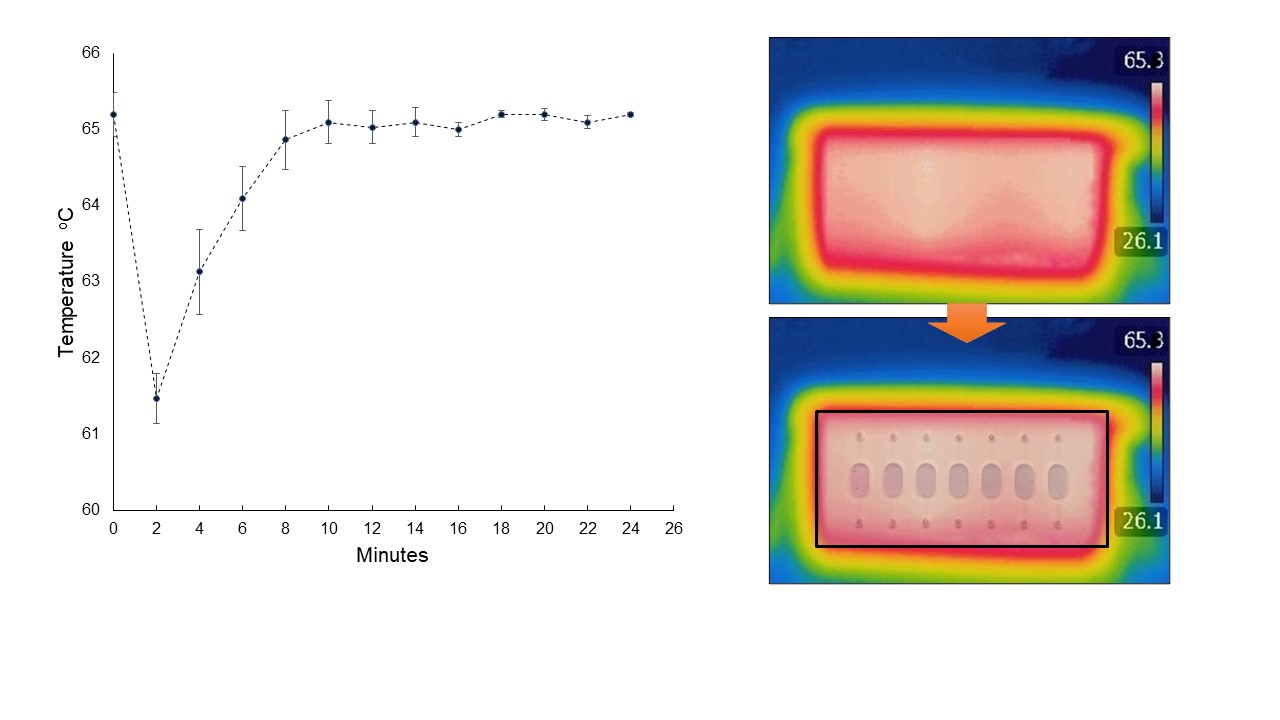


**Fig. S5**. The temperature distribution on the chip, which was measured by a FLIR camera.

**Table S1.** AIC score of the various fitting models. The 7-parameters sigmoidal model has the lowest score in 14 out of 18 datasets. (Chamber name: chamber number_experimental chip (1 to 3))

| **Chamber name** | **4-parameters** | **5-parameters** | **6-parameters** | **7-parameters** |
| --- | --- | --- | --- | --- |
| C1_1 | -421.76 | -430.36 | -432.02 | -447.58 |
| C1_2 | -413.38 | -424.52 | -428 | -442.5 |
| C1_3 | -418.63 | -431.46 | -440.92 | -455.08 |
| C2_1 | -425.59 | -439.74 | -441.29 | -454.81 |
| C2_2 | -427.69 | -439.92 | -444.46 | -452.01 |
| C2_3 | -429.88 | -443.88 | -451.67 | -460.45 |
| C3_1 | -409.27 | -430.1 | -435.74 | -444.25 |
| C3_2 | -414.8 | -429.45 | -445.07 | -448.84 |
| C3_3 | -419.37 | -432.22 | -452.85 | -456.38 |
| C4_1 | -398.76 | -424.65 | -439.4 | -440.68 |
| C4_2 | -410.47 | -421.37 | -443.75 | -442.81 |
| C4_3 | -415.69 | -428.51 | -455.45 | -453.83 |
| C5_1 | -396.12 | -423.8 | -445.85 | -444.58 |
| C5_2 | -412.18 | -423.79 | -450.35 | -448.9 |
| C5_3 | -415.23 | -425.25 | -459.84 | -461.06 |
| C6_1 | -394.97 | -408.91 | -434.67 | -449.49 |
| C6_2 | -405.3 | -409.1 | -438.65 | -452.11 |
| C6_3 | -410.01 | -411.49 | -445.83 | -462.05 |

(Chamber name: chamber number_experimental chip (1 to 3))

**Table S2.** AIC weight core of the various fitting models. The 7-parameters sigmoidal model AIC weight score shows the closest to 1 in 14 out of 18 datasets.

| **Chamber name** | **4-parameters** | **5-parameters** | **6-parameters** | **7-parameters** |
| --- | --- | --- | --- | --- |
| C1_1 | 0 | 0 | 0 | 0.999 |
| C1_2 | 0 | 0 | 0.001 | 0.999 |
| C1_3 | 0 | 0 | 0.001 | 0.999 |
| C2_1 | 0 | 0.001 | 0.001 | 0.998 |
| C2_2 | 0 | 0.002 | 0.022 | 0.975 |
| C2_3 | 0 | 0 | 0.012 | 0.988 |
| C3_1 | 0 | 0.001 | 0.014 | 0.985 |
| C3_2 | 0 | 0 | 0.132 | 0.868 |
| C3_3 | 0 | 0 | 0.146 | 0.854 |
| C4_1 | 0 | 0 | 0.345 | 0.655 |
| C4_2 | 0 | 0 | 0.615 | 0.385 |
| C4_3 | 0 | 0 | 0.692 | 0.308 |
| C5_1 | 0 | 0 | 0.654 | 0.346 |
| C5_2 | 0 | 0 | 0.674 | 0.326 |
| C5_3 | 0 | 0 | 0.352 | 0.648 |
| C6_1 | 0 | 0 | 0.001 | 0.999 |
| C6_2 | 0 | 0 | 0.001 | 0.999 |
| C6_3 | 0 | 0 | 0 | 1 |

(Chamber name: chamber number_experimental chip (1 to 3))

**Table S3.** Primer sequence information for the LAMP reactions

| Target gene  (Accession #) | Primer | Sequence (5’ to 3’) | Length [bp] |
| --- | --- | --- | --- |
| *E. coli W*  fliC gene  (ATCC 9637) | FIP | CAGTCCCGTATTCAGGACGCCGGATGATCTGCGCTTTCGA | 40 |
|  | BIP | GGTGACTGCGGAATCCAGACGTGAGGCAATTGCATCCATCG | 41 |
|  | F3 | GCCAACACGGAGTTACCG | 18 |
|  | B3 | GCTCTGCAACCAAAGATCCA | 20 |
| *S. Typhimurium*  invA gene  (M90846.1) | FIP | TCGACTCCACATTCACTCGATTACTGATAACTTGCCAGACG | 41 |
|  | BIP | CCTGCCGAATGGCGATTACAATTAATTGTCGATTCAGCCG | 40 |
|  | F3 | CAACCATGGTGACTGTGA | 18 |
|  | B3 | CCAGCGACCTTTCTCTGA | 18 |
| *V. parahaemolyticus*  ropD gene  (ATCC 17802) | FIP | GCAATAAACGATTTCTTCGGCATTTCAAGAACGTTTGATCATGAAGT | 49 |
|  | BIP | CCTATTCACTGGTACGAATCAAGTGTTTGATTTTCTCAGCGTATGG | 48 |
|  | F3 | GTACAGCAATGGATCGCG | 20 |
|  | B3 | CGACGGATCTCTTCTTCG | 20 |

**Experiments for the direct LAMP reaction**

*E. coli* cells were cultured in an LB broth, collected by centrifugation at 13,000 rpm for 10 min, and resuspended in ddH_2_O. A NanoDrop 3000 (Thermo Scientific) was used to estimate the concentration of the bacteria cells based on the optical density at 600 nm. The 10-fold serially diluted bacteria (from 1×10^2^ to 1× 10^7^) were prepared to obtain the calibration curve. The direct LAMP reaction was carried out on a chip with a reaction volume of 30 µL. Each reaction chamber contains 3.6 µL of 10× Isothermal Amplification buffer (New England Biolabs, USA), 1.5 µL of 100 mM MgSO_4_ (New England Biolabs, USA), 13.2 µL of 2.5 mM dNTPs (Takara Korea Biomedical Inc, Korea), 0.84 µL of 100 µM F3 primer, 0.84 µL of 100 µM B3 primer, 0.84 µL of 100 µM FIB primer, 0.84 µL of 100 µM BIP primer, 1 µL of 3 mM EBT (Sigma, Germany), 1 µL of a bacterial cell sample, 1.24 µL of 5M betaine (Sigma-Aldrich, USA), 3.75 µL of 8000U Bst Polymerase 2.0 (New England Biolabs, USA) and 5.1 µL of EZ-way buffer (Komabiotech, Korea). The LAMP box was powered for 10 min in advance for pre-heating the chip-holding site to reach 65 ^o^C. The LAMP reaction was completed at 65 °C using the i-Genbox platform.

**Calibration curve from the direct LAMP reaction**

The direct LAMP reaction does not require any sample preparation steps, such as cell lysis, DNA extraction and purification to amplify specific target genes. Thus, it is more adequate for point-of-care genetic analysis to identify the pathogens. For the quantification of bacterial cells, it is necessary to obtain the calibration curve using the serially diluted bacteria samples. As shown in Fig. S6A, as the input cell number increased, the color changed faster. Compared with the purified DNA templates, the direct LAMP reaction took longer. The threshold time of the bacterial concentration of 1×10^7^ was 47.23 min, while that of 1×10^2^ was 76.74 min. In a similar way for the DNA quantification, we could generate a calibration curve on a smartphone, which displayed a nice correlation between the threshold time and the bacterial cell number (Fig. S6B).


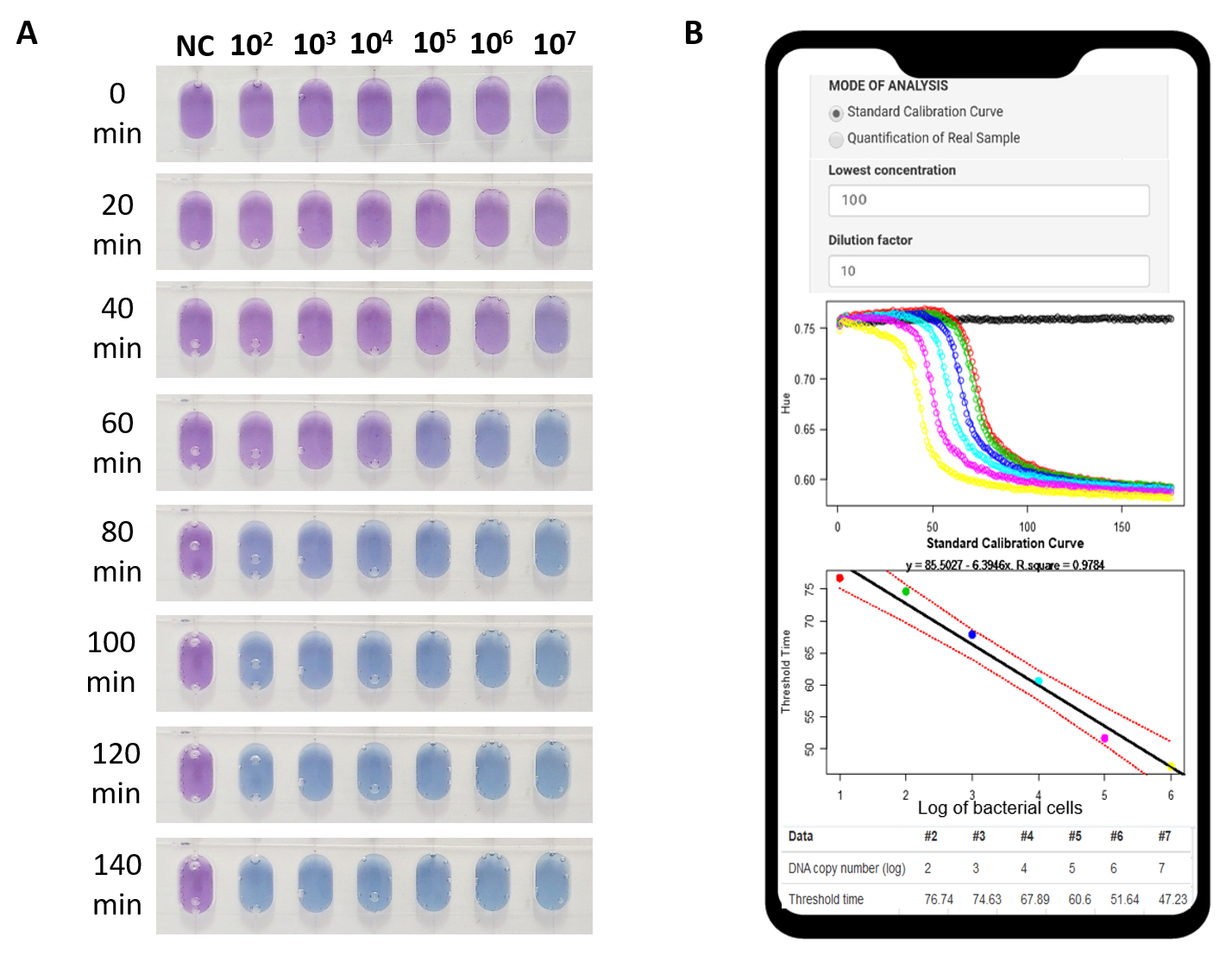


**Fig. S6**. Direct quantification of E. coli cells. (A) Digital images of the direct LAMP reaction. The chamber containing high cell numbers changed the color from purple to blue faster. (B) With the mode of ‘Standard Calibration Curve’, the serially diluted samples generated typical qLAMP profiles and the threshold value, which is determined by the second derivative maximum, revealed the threshold time for each diluted sample. Thus, the standard curve plotting the threshold time versus the logarithm of bacterial cell number can be generated. The black line represents the linear fit to the data, and the red lines indicate the 95% confidence interval.
